# Clinical or Imaging Diagnosis of the Current Medical Practice for Superior Vena Cava Syndrome?

**DOI:** 10.3390/diagnostics11112058

**Published:** 2021-11-06

**Authors:** Liliana Dragomir, Virginia Marina, Mihaela Anghele, Aurelian-Dumitrache Anghele

**Affiliations:** 1 Clinical-Medical Department, Faculty of Medicine and Pharmacy, “Dunarea de Jos” University of Galati, 800201 Galati, Romania; lilianadragomir2017@gmail.com; 2 Medical Department of Occupational Health, Faculty of Medicine and Pharmacy, “Dunarea de Jos” University of Galati, 800201 Galati, Romania; 3 Medical Department, Faculty of Medicine and Pharmacy, “Dunarea de Jos” University of Galati, 800201 Galati, Romania; Mihaela.Anghele@ugal.ro; 4 Department of General Surgery, Faculty of Medicine and Pharmacy, “Dunarea de Jos” University of Galati, 800201 Galati, Romania; anghele_aurelian@yahoo.com

**Keywords:** superiorvena cava syndrome, clinical diagnosis

## Abstract

Most cases of superior vena cava syndrome are easily diagnosed bya clinical examination alone, but several diagnostic tests and procedures can be helpful. When a patient presentswith a suspected diagnosis of superior vena cava syndrome, the first step is to obtain an imaging study that confirms the diagnosis and aids treatment decisions. Magnetic resonance imaging, contrast-enhanced CT scanning, radionuclide flow studies and traditional venography are all appropriate techniques. Still, the CT scan is the most readily available technology in most centers. The CT scan and magnetic resonance imaging also provide information on possible etiologies and can therefore direct the approach towards a tissue diagnosis.

## 1. Introduction

Superior vena cava syndrome is represented by the partial or the complete obstruction of the superior vena cava into its course through the superior mediastinum. 

**Figure 1:**
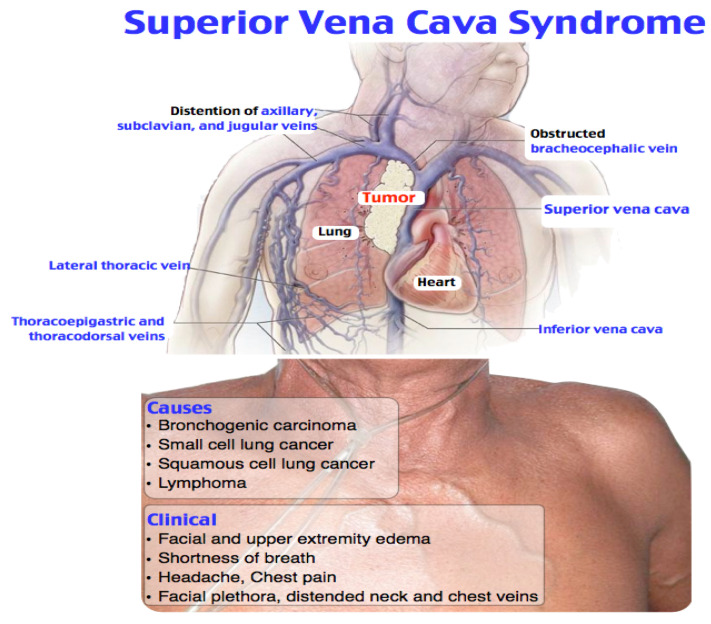
The syndrome was originally described as being secondary to an infection such as tuberculosis or a syphilitic aortic aneurysm [1,2,3]. Currently, superior vena cava syndrome is generally due to cancer or thrombotic events.

The venous obstruction may be due to the compression, invasion, thrombosis, or fibrosis of the superior vena cava. Secondary to the obstruction, there is an increase in central venous pressure and the appearance of collateral circulation, in addition to frequent shunting through the azygos vein system, leading to the signs and the pathognomonic symptoms for this pathology. The collateral venous network, predominant on the anterior aspect of the thorax, is sometimes accompanied by cyanosis and the “cape” oedema (Figure 1).

The lungs, breast and mediastinal neoplasms are common causes of superior vena cava syndrome [2,4,5,6,7]; lung adenocarcinoma is the most common cause [1,2,8] (Table 1).

Most cases of superior vena cava syndrome are easily diagnosed by a clinical examination alone; however, several diagnostic tests and procedures can be helpful. When a patient presents with a suspected diagnosis of superior vena cava syndrome, the first step is to obtain an imaging study that confirms the diagnosis and aids in treatment decisions. Magnetic resonance imaging, contrast-enhanced CT scanning, radionuclide flow studies and traditional venography are all appropriate techniques. Still, the CT scan is the most readily available technology in most centers. The CT scan and magnetic resonance imaging also provide information regarding possible etiologies and can therefore direct the approach towards a tissue diagnosis. The approach to establishing a tissue diagnosis is defined by both the clinical stability of the patient and the findings of the clinical examination and radiographic studies. The tissue diagnoses are important because they guide the treatment specifically, identifying the patients for whom superior vena cava syndrome should be treated using combined chemotherapy rather than local measures such as radiotherapy and percutaneous vascular procedures.

The treatment of superior vena cava syndrome is divided into supportive and definitive therapy.

Depending on the etiological factors, the tumor and the infection, specific drugs or radiation may be used [1]. Patients with superior vena cava syndrome usually have the advanced disease, and less than 10% survive longerthan 30 months after the treatment [4].

## 2. Case Presentations

We present the case of a male 54-year-old patient diagnosed with superior vena cava syndrome brought by ambulance to the emergency room withthe following symptoms: exceptional dyspnea, with a progressive onset over several days; physical asthenia, at low effort; dysphagia for solid food; a dry cough; dizziness; the cyanosis of the cephalic extremity, the anterior cervical region and the anterosuperior thorax.

In addition to his personal pathological history, he was monitored by the cardiology service for the following symptoms: ischemic cardiomyopathy with a significant left ventricular systolic dysfunction; old anteroseptal myocardial infarction; the second degrade mitral insufficiency; mild aortic insufficiency; medium pulmonary hypertension; NYHA class III; congestive heart failure with low ejection fraction; dyslipidaemia; steatohepatitis; dietary non-compliance to chronic treatment, e.g., a loop diuretic (furosemide), a potassium-sparing diuretic (spironolactone), beta-blockers (metoprolol), cardiac glycoside (digoxin) and an antiplatelet (aspirin). The last admission to the Cardiology Department was for the following clinical symptoms: acute pulmonary oedema due to a lack of dietary compliance; a respiratory infection with a favorable evolution; discharge without angina; no congestion in sinus rhythm (EKG—sinus tachycardia, QS aspect from V1–V5).

**Figure 2:**
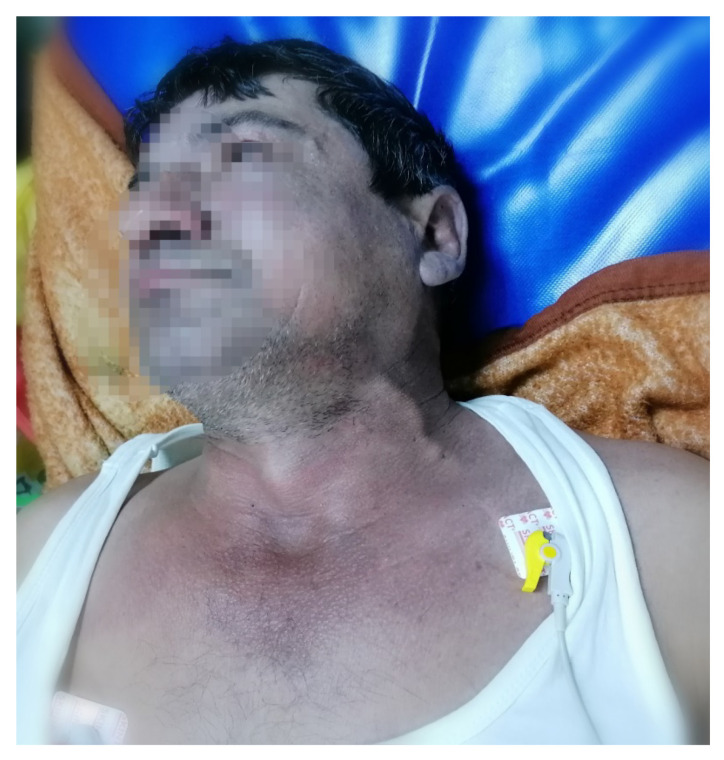
The patient’s turgid jugular.

To the current presentation, two months after the last admission in December 2020, the physical examination (Figure 2) revealed: the general condition influenced the conscious and cooperative patient; moderate dyspnoea on the exertion cyanosis of the cephalic extremity, thecervical region and upper 1/3 of the chest; gambit oedema.

The monitoring of vital parameters afterthe admission to hospital provided the following information: Glasgow score = 15 points, respiratory rate = 12 breaths/minute, ventricular rate = 100 beats/minute, blood pressure = 120/80 mmHg, SpO_2_ 98% in atmospheric air, temperature = 36.2 °C.

**Figure 3:**
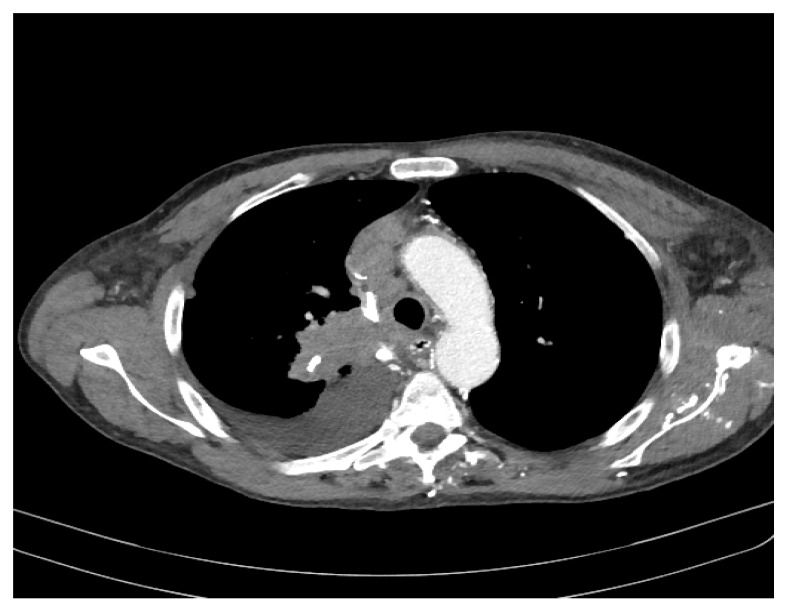
Computer tomography of the patient.

**Figure 4:**
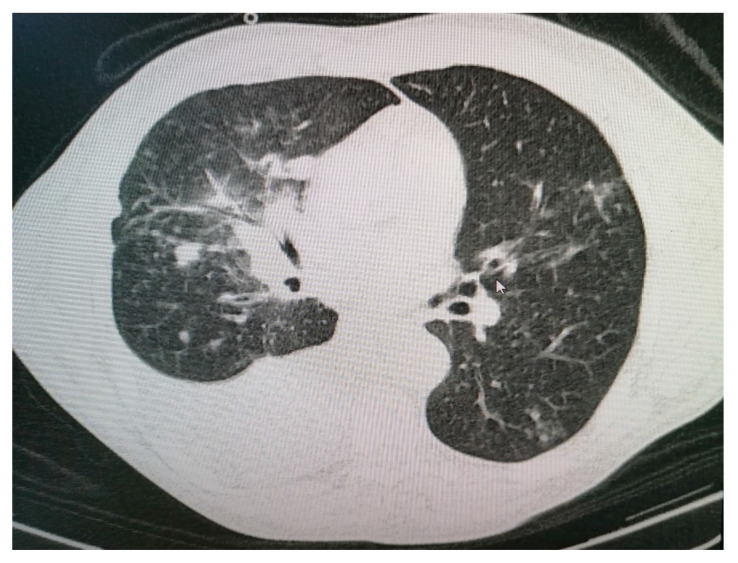
Computer tomography of the patient.

At this time, the suspicion of mediastinal compression syndrome is raised, which is why it is recommended, in addition to the biological investigations, to perform a chest CT with intravenous contrast, which showed the following changes: to the right upper lobe of the paratracheal level, a solid, non-homogeneous, moderately iodophilic tumor formation was observed, which touches upon the trachea, the right main bronchus, the right upper lobar bronchus and, partially, the right pulmonary artery. The right main bronchus is slightly compressed by the development of the tumor around it (Figure 3 and Figure 4). The described formation also insinuates posteriorly to the right pulmonary artery. Between the inferior bronchus and the left upper lobar bronchus, there are several nodules, with diameters between 4 and 13 mm, visible subpleurally but also to the intrapulmonary inferior level of the described tumor.

**Figure 5:**
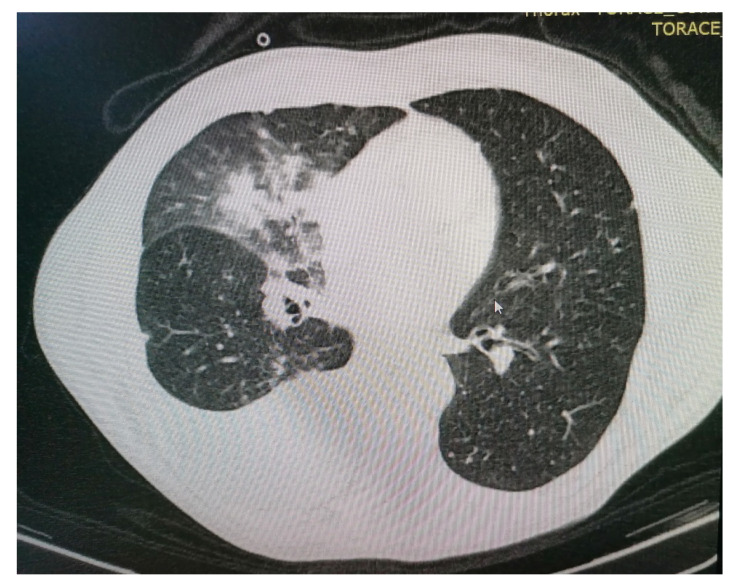
Computer tomography of the patient.

We observed a fibrous, sequelae-like plaque which is visible antero-basal right paracardiac; the right pleura with the medium amount; the volume of the left heart cavities aregreatly enlarged (Figure 5).

**Figure 6:**
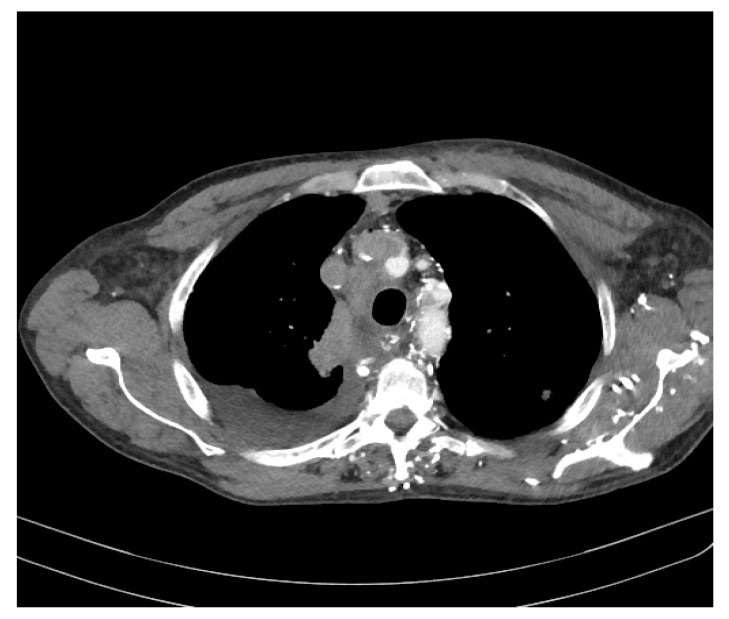
Computer tomography of the patient.

The brachiocephalic venous trunk is inhomogeneously opacified with the intravenous contrast (roughly 1/3 of the lumen is opacified with intravenous contrast), and the superior vena cava is almost neopacified with only a discrete peripheral contrast loading ring. The following can be observed: the presence of collateral circulation in the cervical region and the left upper limb; left apical fibro-nodular sequelae; a small fibro-nodular plaque located basally on the left anterolateral side (Figure 6).

**Figure 7:**
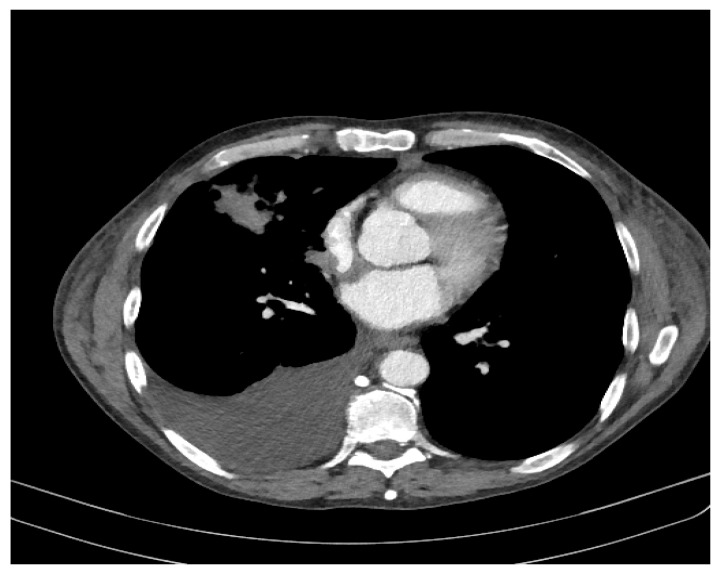
Computer tomography of the patient.

We observed (Figure 7) the liver with an increased size, clear outline, homogeneous structure and no focal lesions. The portal vein is 9 mm and is permeable. The main bile ducts and intrahepatic bile ducts are undiluted. The gall bladder is small in volume, without stones. The spleen and pancreas are present a normal CTscan appearance. Both the adrenal glands show nodules with solid consistency, susceptible to the secondary determinations (on the right about 8/14 mm, on the left about 14/18 mm).

Based on the clinical examination in conjunction with the biological and imaging investigations, the diagnosis of the right upper lobe bronchopulmonary tumor with secondary pulmonary and mediastinal findings, superior vena cava syndrome was established.

A biopsy was taken for the histological examination, pharmacological therapy (Dexamethasone, antibiotic therapy and the treatment of cardiac diseases) and radiotherapy. The decompressive purpose, at the mediastinal level, was initiated with a favorable evolution until now.

## 3. Discussions

Superior vena cava syndrome was first ascribed by William Hunter in 1757 to a patient with a large syphilitic aortic aneurysm compressing the superior vena cava. It occurs in roughly 15,000 people in the United States each year [4].

More than 80% of superior vena cava syndrome cases are caused by malignant mediastinal tumors, which cause the invasion of the venous intima or an extrinsic mass effect [9,10,11].

The most common malignant cause is small cell lung cancer in about 50% of patients.

When managing superior vena cava syndrome, the goals are to relieve the symptoms and attempt to cure the primary malignant process. Only a small percentage of patients with rapid-onset superior vena cava obstruction are at the risk of life-threatening complications [12].

Patients with superior vena cava syndrome often achieve significant symptomatic improvement from the conservative treatment measures, including the bedhead elevation and supplemental oxygen administration [13]. The emergency treatment is indicated when a cerebral oedema, decreased cardiac output or an upper airway oedema is present. The corticosteroids and diuretics treatmentsare often used to relieve the laryngeal or cerebral oedema, although the documentation of their efficacy is questionable.

The radiotherapy treatment has been advocated as a standard treatment for most patients with superior vena cava syndrome. This is used as an initial treatment if a histological diagnosis cannot be established and the patient’s clinical condition deteriorates; however, reviews suggest that superior vena cava syndrome obstruction alone is rarely an absolute emergency requiring the treatment without a specific diagnosis [14,15,16]. 

The goals of pharmacotherapy are to reduce morbidity and to prevent complications.

## 4. Conclusions

Our patient presented the classic signs and symptoms of superior vena cava syndrome, including dyspnea (which is the most common symptom reported in the specialized literature), jugular distention (exacerbated by leaning forward or hyperextension of the head) and a cough (secondary to the upper airway functional compromise).

Superior vena cava syndrome is most commonly seen in patients with a malignant disease, especially in those with lung cancer. 

Clinically, the patients frequently presentshortness of breath, associated with facial oedema and cyanosis of the upper extremity.

In the case of tumor pathology, since the obstruction has occurred slowly over time, it is preferable tofirstobtain a histological diagnosis before starting the treatment. 

The emergency treatment is indicated when cerebral oedema, decreased cardiac output or upper airway oedema is present.

Radiotherapy, with or without chemotherapy, is the mainstay of treatment for most patients. Due to the poor overall prognosis of most patients with malignant tumors, palliation is often the focus of treatment.

The particularity of our case is that we were able to diagnose the presence of superior vena cava syndrome clinically.

The patient had symptoms as in the semiology books. We have confirmed our clinical diagnosis of the superior vena cava syndrome using current medical technology. However, our clinical diagnosis of superior vena cava syndrome was a secondary diagnosis due to a right upper lobe bronchopulmonary tumor with secondary pulmonary and mediastinal findings.

The assessment of the severity of the fragile syndrome of a patient admitted to the Emergency Department is made, in the first instance, on the basis of clinical examination and anamnestic data. The diagnosis of certainty will be made on the basis of these data but also on the basis of imaging investigations. In our case, emergency medicine specialists, the scales used routinely are GCS (Glasgow Coma Scale) and RTS (Revised Trauma Score). Each of the other medical or surgical specialties hasits own scores to assess the severity and prognosis of a pathology. We mention that we work in a lower-level hospital, where we do not have resources for all medical and surgical specialties.This is necessary when the complexity of a case exceeds the diagnostic and treatment capacity of the hospital; therefore, in these cases, we must transfer patients to higher-level centers specializing in different pathologies.

## Figures and Tables

**Table 1:** Common causes of superior vena cava syndrome.

Malignant (>85%)	Benign (3% to 15%)
Lung cancer	Indwelling catheters
Lymphoma	Thymoma
Breast cancer	Cystic hygroma
	Tuberculosis
	Histoplasmosis
	Thyroid goiter
	Aortic aneurysm

## Data Availability

Informed consent was obtained from all subjects involved in the study.
